# Summer Diarrhœa

**Published:** 1892-08

**Authors:** W. N. Rogers

**Affiliations:** Belton, Texas


For Daniel's Texas Medical Journal.

### SUMMER DIARRHŒA.

W. N. ROGERS, M. D., BELTON, TEXAS.

AS THIS is the season for bowel diseases among teething children, and as I find that these diseases are so often treated in an unscientific way, I wish to say a few words in their behalf. I find by actual observation that the majority of our practicing physicians are still treating these summer diarrhœas by the old method of astringents and opium, instead of the recent and scientific plan of intestinal antisepsis. It is surprising the number of physicians who are considered progressive, that are now ten years behind in this respect. There is no more comparison between the old plan of opium, astringents, and chalk, and the present plan of antiseptic treatment of diarrhœa that there is between the old method of dressing wounds and the present plan where blood poisoning is absolutely prohibited.

About five years ago I published an article in the *Texas Courier of Medicine* in which I gave my plan of treatment and its success. I did not then, nor do I now claim originality in the treatment of these diseases, but that I was keeping pace with the advance in the treatment of this class of disorders. I was only on the side of scientific medicine. Since then I have watched every improvement that has been made in this line, and I am safe in saying that if the present antiseptic methods are rigidly carried out in connection with modern dietary rules, that very few children will die of summer diarrhœa. I can say the same thing in reference to typhoid fever, dysentery, and diarrhœa of all kinds. I have had opposition of the most bitter kind from the old fogy doctors who sit in the front door of the drug store all day long and depend on their funny anecdotes to gain practice for them, but I care not for this, if I can look over my list of patients for the past eight years and see success has been on my side.

Recently, a case came to my knowledge where chalk and salacin, combined with astringents, were relied on by the physician. And the more absurd part was the refusal of water when the little patient was crying for it. While in New York this summer, I observed that the hospital physicians laid great stress on plenty of good pure water in connection with antiseptic medicines and careful feeding.

There are a number of drugs that will accomplish the desired object, and it depends on one's own notions as to which is the best. My favorite prescription is a combination of bismuth, salicylate of soda, salol, and, where indicated, some preparation of opium. I have had such uniform success with this that I have not tried very entensively any other. Probably naphthalin, salicylate of bismuth, and many others are good, as many of our authorities claim. I frequently combine small quantities of calomel with the first day or two's treatment if indicated.

It is a settled fact that fermentation is at the bottom of these disorders, and if carried out thoroughly these drugs will arrest this process.

A good plan to begin with is to clean out the bowels first with castor oil. There will occasionally be a case that will not digest the medicine, owing to the extreme irritation of the stomach, and in such cases the chances of success are very bad, but otherwise we can promise good results, as a rule, if proper feeding and medication are observed. It is in the interest of these little sufferers I write, and beg a fair chance for their life, by giving them the benefit of scientific treatment.

If this treatment is rigidly observed, in from twenty-four to forty-eight hours, all that offensive odor, tympanites, etc., will disappear, and then you have the case under control.

